# Zwitterionic Hydrogel Activates Autophagy to Promote Extracellular Matrix Remodeling for Improved Pressure Ulcer Healing

**DOI:** 10.3389/fbioe.2021.740863

**Published:** 2021-10-08

**Authors:** Yuan Li, Shishuang Jiang, Liwan Song, Zhe Yao, Junwen Zhang, Kangning Wang, Liping Jiang, Huacheng He, Cai Lin, Jiang Wu

**Affiliations:** ^1^ School of Pharmaceutical Sciences, Key Laboratory of Biotechnology and Pharmaceutical Engineering, Wenzhou Medical University, Wenzhou, China; ^2^ Department of Burn, The First Affiliated Hospital of Wenzhou Medical University, Wenzhou, China; ^3^ College of Chemistry and Materials Engineering, Wenzhou University, Wenzhou, China

**Keywords:** pressure ulcer, zwitterionic, non-fouling, hydrogel, autophagy

## Abstract

Pressure ulcer (PU) is a worldwide problem that is hard to heal because of its prolonged inflammatory response and impaired ECM deposition caused by local hypoxia and repeated ischemia/reperfusion. Our previous study discovered that the non-fouling zwitterionic sulfated poly (sulfobetaine methacrylate) (SBMA) hydrogel can improve PU healing with rapid ECM rebuilding. However, the mechanism of the SBMA hydrogel in promoting ECM rebuilding is unclear. Therefore, in this work, the impact of the SBMA hydrogel on ECM reconstruction is comprehensively studied, and the underlying mechanism is intensively investigated in a rat PU model. The *in vivo* data demonstrate that compared to the PEG hydrogel, the SBMA hydrogel enhances the ECM remolding by the upregulation of fibronectin and laminin expression as well as the inhibition of MMP-2. Further investigation reveals that the decreased MMP-2 expression of zwitterionic SBMA hydrogel treatment is due to the activation of autophagy through the inhibited PI3K/Akt/mTOR signaling pathway and reduced inflammation. The association of autophagy with ECM remodeling may provide a way in guiding the design of biomaterial-based wound dressing for chronic wound repair.

## Introduction

Pressure ulcer (PU), also known as pressure injury (PI), refers to localized injury of the skin and the potential subcutaneous soft tissue created by severe and/or continuous pressure or pressure combined with a shear force (Whitney et al., 2006; Edsberg et al., 2016). PU now remains the most common chronic wound healing problem in elderly, spinal cord injured, and immobilized patients. With the aging population and extension of expected life, patients with PU are continuously increasing, and it has become a serious therapeutic challenge in multiple countries (Gorecki et al., 2009), especially with stage III or IV PU, more than 30% of the patients failed to heal even with 2-year treatment (Brandeis et al., 1990), which not only results in bad quality of life but also leads to patients’ death (Landi et al., 2007). Chronic wounds like PU usually involve a delayed healing process due to the complex situation of wound microenvironment such as excessive inflammation reaction and impaired ECM deposition as well as inhibited vascularization caused by local hypoxia and repeated ischemia/reperfusion (I/R) (Thomas Mustoe, 2004; Mustoe et al., 2006). To accelerate the recovery of PU, various drugs have been promoted, such as siRNA (Li et al., 2017; O’Rourke et al., 2019), DNA (Tokatlian et al., 2015; Rabiee Motmaen et al., 2020), gasotransmitters (Liu et al., 2014; Wu et al., 2016a), growth factors (Wu et al., 2016b; Wu et al., 201c; Huang et al., 2019), and stem cells (Chen et al., 2019; Wang X et al., 2019). However, local administration alone is often difficult to exert the effect of the drugs fully and permanently. Advances in biomaterials such as nanoparticles (Hasan et al., 2019; Nguyen et al., 2019; Wu et al., 2021), hydrogels (Wu et al., 2018), films (Chen Y et al., 2020), scaffolds (De Angelis et al., 2019; Wang S et al., 2019), and gauzes (Li et al., 2020; Xiao et al., 2020) make it possible to synthesize effective delivery systems for PU treatment.

**GRAPHICAL ABSTRACT:**
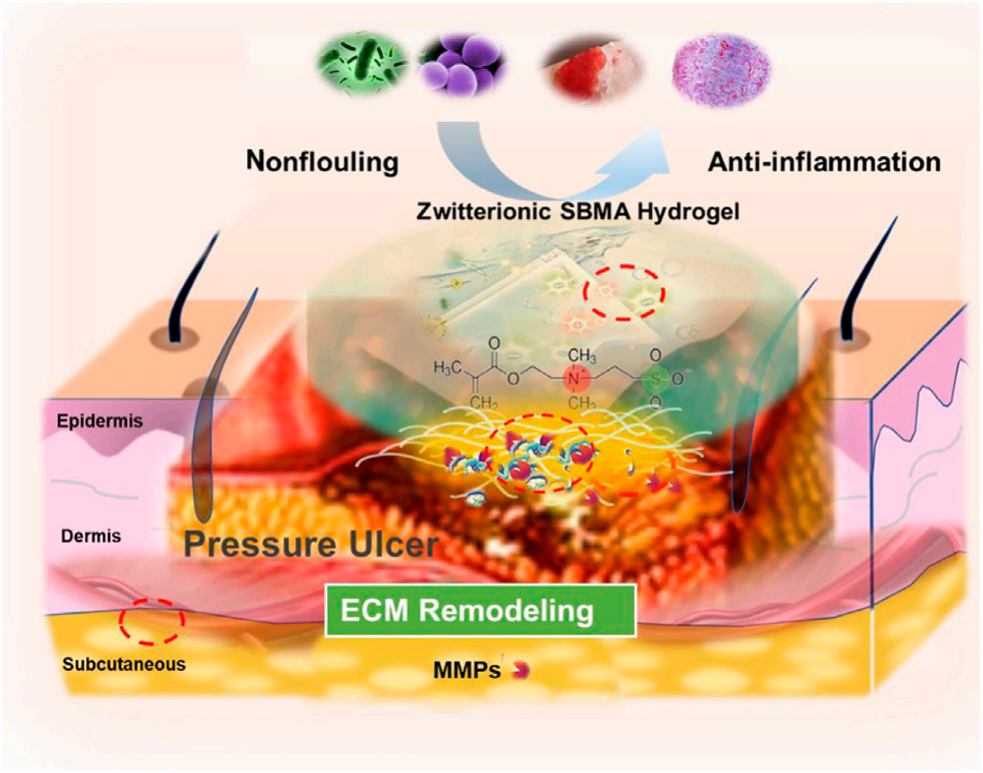
xxx

Compared to traditional biomaterials, hydrogels as wet dressings have attracted increasing attention for the treatment of pressure ulcers (Kirker et al., 2002; Gong et al., 2013; Chen A et al., 2020). Maintaining the wet environment of the wound is a significant method to treat PU. The moisture in the wound promotes the natural autolysis process that breaks down undesired wound components, such as necrotic tissue, thereby reducing the inflammation reaction (M, 2008). Among all these hydrogel systems, PEG-based hydrogels are widely used because of the excellent property of biocompatibility (Veronese and Pasut, 2005). However, another non-fouling material zwitterionic poly (sulfobetaine methacrylate) (pSBMA) is considered as an alternative biomaterial to PEG due to its superior thermal stability, biological compatibility, low immune response, and anti-protein adhesion ability (Keefe and Jiang, 2011; Zhang et al., 2013; Xiao et al., 2021). Our previous reports have shown that zwitterionic SBMA hydrogels are superior to PEG hydrogels in promoting wound healing since the SBMA hydrogels can stimulate re-epithelization and neovascularization, trigger macrophage polarization, and perform better in controlling drug release (Wu et al., 2018; He et al., 2019; Xiao et al., 2021). More interestingly, in comparison with the PEG hydrogel, we discover that the SBMA hydrogel exhibits better deposition of collagen with much well-aligned collagen fibers in both acute wounds and chronic wounds (e.g., PU) (Jiang et al., 2018; Wu et al., 2018). As collagen is one of the main components in extracellular matrix (ECM), the well-deposited collagen should highly contribute to the ECM rebuilding in the wound bed. The well-constructed ECM eventually provides a favorable environment for many cell activities (e.g., proliferation and migration), which contributes to the rapid wound healing (Wysocki and Grinnell, 1990; Singer and Clark, 1999; Cox and Erler, 2011; Sackey-Aboagye et al., 2016). The successful ECM reconstruction in PU by the SBMA hydrogel is especially important since the ECM is extremely destroyed by the pressure in PU and normally hard to be recovered (Norgauer et al., 2002). Based on the above analysis, we highly believe that the SBMA hydrogel plays a key role in rebuilding the ECM in the wound, which finally induces the rapid wound repair. Nevertheless, the underlying mechanism of the SBMA hydrogel in promoting the ECM reconstruction remains elusive and has not been fully elucidated.

Therefore, herein, we have comprehensively assessed the ECM remodeling after the SBMA hydrogel treatment in a rat PU model, which validates that the SBMA hydrogel can effectively rebuild the ECM in the PU wound bed. Furthermore, the underlying mechanism of the SBMA hydrogel for rapid ECM rebuilding is also intensively investigated. The data reveal that the downregulation of MMP-2 expression after the treatment of the SBMA hydrogel is key to the ECM reconstruction. Further study demonstrates that the SBMA hydrogel regulates the MMP-2 expression by activating the autophagy through the inhibition of the PI3K/Akt/mTOR signaling pathway. The discovery of the autophagy involved in the ECM construction in chronic wounds (e.g., PU) may provide a way in guiding the design of biomaterial-based wound dressing in the near future.

## Materials and Methods

### Materials and Reagents

[2-(Methacryloyloxy)ethyl]dimethyl-(3-sulfopropyl) ammonium hydroxide (SBMA), 2-hydroxy-4-(2-hydroxyethoxy)-2-methylpropiophenone (98%), and poly (ethylene glycol) dimethacrylate (PEGDMA, MW = 550 Da) were obtained from Sigma-Aldrich (MO, USA). Poly (ethylene glycol) methyl ether methacrylate (PEG, MW = 475 Da) was obtained from Aladdin (Shanghai, China). A hematoxylin–eosin (H&E) stain kit, BCA assay kit, and Masson’s trichrome (MTS) stain kit were purchased from Beyotime (Beijing, China). Collagen I (ab21286), collagen III (ab7778), laminin (ab11575), fibronectin (ab268020), CD31 (ab28364), VEGF (ab39256), CD68 (ab955), CD163 (ab182422), sQSTM1/p62 (ab56416), TNF-α (ab9755), goat anti-rabbit IgG Alexa Fluor® 647 (ab150083), and goat anti-mouse IgG Alexa Fluor 488® (ab150113) were obtained from Abcam (CA, UK). Matrix metallopeptidase2 (MMP-2) (384993) and laminin (383353) were obtained from Zenbio (Chengdu, China). Microtubule-associated one protein light chain 3 (LC3, L7543) was purchased from Sigma-Aldrich. PI3K (4228), p-PI3K (4257), AKT (4691), p-AKT (4060), mTOR (2983), and p-mTOR (5536) were obtained from Cell Signaling Technologies (Beverly, MA, USA). The RIPA lysis buffer was purchased from GE Healthcare Biosciences (PA, USA). The DAB Chromogen Kit was purchased from ZSGB-BIO (Beijing, China).

### Preparation of the SBMA and PEG Hydrogels

The SBMA hydrogel was prepared according to our previous report (Wu et al., 2018). Briefly, sulfobetaine methacrylate (SBMA, 2.8 g) was dissolved in 2 ml ultrasonically degassed distilled water. Under UV light conditions, the cross-linking agent PEGDMA and an initiator with a weight of 0.1% monomer were added. Then, the mixture was placed into two glass slides, separated by a Teflon plate with a thickness of 1 mm and irradiated with a 362-nm ultraviolet lamp SB-100P (Spectroline, NY, USA) for 10 min for hydrogel polymerization. Next, the hydrogels were immersed in distilled water for more than 3 days, during which the distilled water was changed daily to remove the unreacted chemicals. Finally, the hydrogels were cut into the disk-like shapes with a diameter of 10 mm for further application. For the preparation of PEG hydrogel, PEG methyl ether methacrylate (PEG, MW = 475 Da) was used as the monomer. All other procedures were the same as those of the SBMA hydrogel.

### Equilibrium Water Content

Hydrogel’s EWC was evaluated by weighting the difference between a fully hydrated hydrogel in saline (NS) solution and the same one which was fully dried. To acquire the EWC of SBMA and PEG hydrogels at equilibrium, the lyophilized hydrogel was incubated in a saline (NS) solution overnight at room temperature. The water adsorbed on the hydrogel surface was removed with a filter paper, and the hydrogel was immediately weighed on an electronic balance, and the wet weight of the hydrogel was measured as well. EWC is determined by the following formula:

$$\text{EWC}\left(\%\right)=\left[\left({\text{W}}_{\text{S}}-{\text{W}}_{\text{d}}\right)/{\text{W}}_{\text{S}}\right]\times 100\%.$$

EWC is the percentage of water the hydrogel has at equilibrium. W_S_ represents the weight of water absorbed by the hydrogel at equilibrium, and W_d_ represents the lyophilized weight of the hydrogel. Each experiment was repeated thrice.

### Measurement of Contact Angle

The water contact angle (WCA) was measured to evaluate the hydrophilicity of the PEG and SBMA hydrogels using a contact angle instrument (Kruss DSA100 Mobiledrop, Germany). The hydrogels were immersed in water to reach the swelling equilibrium (3 days). 5 μl of deionized water was dropped onto the surfaces of the hydrogel by using a microsyringe, and a magnified image of the droplet was obtained using a digital camera. After the removal of the surface water, the contact angle of the PEG and SBMA hydrogels was determined at room temperature with a contact angle goniometer. The result was calculated as average values of three parallel samples.

### Mechanical Properties

The mechanical properties of the SBMA and PEG hydrogels were obtained by compression tests at room temperature using Instron 3595 (Instron Co., MA, USA). Briefly, the hydrogels were cut into disk-like shapes with a diameter of 10 mm and a thickness of 1 mm for the tests. The rate of compression remained constant at 2 mm/min and the experiment stopped when the hydrogel cracked, or the strain of the hydrogel reached to 95%. For each hydrogel, three samples were measured to assess the reproducibility and carry out the statistical analysis.

### Animal Model

Male Sprague Dawley (SD) rats, weighing 300–350 g, were acquired from the Animal Center of Chinese Academy of Sciences (Shanghai, China). Rats were caged separately in the animal laboratory under controlled conditions to optimize animal care. Temperature (22–26°C), humidity (40–60%), and photoperiod (12 h light/dark cycle) were kept constantly. Rats had *ad libitum* access to rodent feed and water under standard laboratory conditions. All procedures were approved by the Animal Experimentation Ethics Committee of Wenzhou Medical University, Wenzhou, China. All animal experiments were performed in accordance with the NIH animal protocol and guideline. The acute wound model was created in SD rats based on the previous report. The PU animal model was created in SD rats with modified procedures based on the previous report (Stadler et al., 2004). In brief, the skin of bilateral gracilis muscle of rats was first shaved, cleaned, and disinfected. Then, the ischemia (I) and reperfusion (R) cycles (I/R cycles) were applied to the rat skins. In brief, both hind legs of the rats were located within two mutually attracted permanent magnets (disk shape, 8-mm in diameter and 4-mm in thickness, and 3500 G) for 12 h to trigger the ischemia (I) process. Thereafter, the magnets were removed for 12 h to induce the reperfusion (R) process. The I/R cycle was repeated four times. After the four I/R cycles, the rats were anesthetized by intraperitoneal injection of 8% chloral hydrate (300 mg/kg). An 8-mm-round skin biopsy perforator was eventually used to remove the injured skins to form the pressure ulcer wounds.

### Treatment of Pressure Ulcers With Hydrogel Dressings

Rats with pressure ulcer wounds were randomly divided into two groups (*n* = 7–8 per group). The SBMA hydrogel or PEG hydrogel (disk-like shape with a diameter of 10 mm) were applied to the wounds. The hydrogel dressings were then covered with a 3M film (3M Healthcare, Germany) and wrapped with a medical bandage. Hydrogel dressings were changed two times per week (once for 3–4 days). On the 4th, 7th, 10th, and 14th days after the initial treatment, the wounds of each group were photographed, and the percentage of wound healing closure was calculated according to the following equation:

$$\text{Wound}\;\text{healing}\;\text{closure}\left(\%\right)=\left[\left({\text{W}}_{1}-{\text{W}}_{2}\right)/{\text{W}}_{1}\right]\times 100\%.$$

where W_1_ represented the wound area on the day of model establishment and W_2_ represented the wound area at the time of observation.

### Histological Analysis

On days 7 and 14, the rats were sacrificed to harvest the wound tissues. The tissues were fixed in cold 4% paraformaldehyde in 0.01 M phosphate-buffered saline (pH 7.4) overnight. Afterward, the tissues were dehydrated and embedded into paraffin. Then, the tissues were cut into 5 μm thickness slices with a microtome (LEICA RM2235, Germany). The tissue slides were finally processed for the hematoxylin and eosin staining (H&E) and Masson’s trichrome staining (MTS). The staining images were obtained with a Nikon light microscope (ECLPSE 80i, Nikon, Japan) for wound healing evaluation. For the quantification of collagen density, the MTS images were split into different colors (red, blue, and green) with the color deconvolution plugin in the Image-Pro Plus 6.0 software. The blue positive area and total staining area were measured by the software, and the collagen density was calculated according to the following equation: Collagen density = (the blue-stained area/the total area) × 100%. Three staining images for each group were selected for the quantification and carry out the statistical analysis.

### Picrosirius Red Staining

Picrosirius red staining was performed following the standard protocols. First, the paraffin sections were soaked in xylene and gradient ethanol for dewaxing and hydration, respectively. Then, the tissue sections were stained with picrosirius red (1 h) and hematoxylin (10 min). Afterward, the skin sections were sealed with neutral resin and observed with a light microscope equipped with polarized light (DM1000, Leica, Germany).

### Immunohistochemical Staining

After deparaffinization and rehydration of skin tissue sections, the endogenous peroxidase activity was blocked with 3% hydrogen peroxide for 15 min at room temperature. After washing with PBS, the nonspecific binding of the samples sites was blocked with 5% BSA for 30 min at 37°C. Subsequently, the sections were incubated at 4°C overnight with antibody against CD31 (1:100), VEGF (1:300), collagen I (1:300), and collagen III (1:300). Afterward, the tissues were washed thrice with PBS and incubated with biotinylated secondary antibodies that diluted with PBS (1:1,000) in 37°C for 1 h. The reaction was stopped by a DAB Chromogen Kit for all sections for 8 s to 3 min, and sections were counterstained with hematoxylin, hydrated, and mounted with a neutral resin. Images were taken using a Nikon light microscope (ECLPSE 80i, Nikon, Japan), and the positive areas of collagen I and collagen III were quantified through Image-Pro Plus 6.0 (Media Cybernetics, Inc., USA).

### Immunofluorescence Staining

Skin sections were de-waxed and hydrated, and the endogenous peroxidase was inactivated with 3% H_2_O_2_, and nonspecific binding of the sections was blocked with 5% BSA. Then, the sections were incubated with antibody against laminin (1:300), fibronectin (1:200), LC3 II (1:200), CD68 (1:200), CD163 (1:200), and TNF-α (1:500) overnight at 4°C. After washing three times with PBS, the sections were incubated with goat anti-rabbit IgG Alexa Fluor® 647 (ab150083) and goat anti-mouse IgG Alexa Fluor 488® (ab150113) (1:1,000 diluted with phosphate-buffered saline) at 37°C for 1 h in the dark. Finally, the tissue sections were stained with DAPI for 5 min and mounted with an anti-fluorescent quencher. The fluorescence images of the tissue sections were taken using a Nikon confocal laser microscope (A1 PLUS, Nikon, Japan), and the images were analyzed by Image-Pro Plus 6.0 software.

### Western Blotting

Tissue samples in each group were obtained from the rat wound area and stored at −80°C before Western blotting. Samples were ground in liquid nitrogen and subsequently homogenized in the RIPA lysis buffer containing protease inhibitor cocktail. The extracts above were collected after centrifugation. Protein concentrations were quantified using the BCA assay. The protein (40 ug) was separated by 7.5–12.5% polyacrylamide gel and then transferred onto PVDF membranes (BioRad Hercules, CA, USA). After blocking with 5% skimmed milk for 2 h at room temperature, the membranes were incubated overnight at 4°C overnight with the following primary antibodies: anti-matrix metallopeptidase2 (MMP-2) (1:1,000), anti-fibronectin (1:1,000), anti-laminin (1:1,000), anti-LC3 (1:1,000), anti-VEGF (1:1,000), anti-CD31 (1:1,000), mouse monoclonal anti-sQSTM1/p62 (1:1,000), PI3K (1:1,000), p-PI3K (1:1,000, 4257), AKT (1:1,000), p-AKT (1:1,000), mTOR (1:1,000), p-mTOR (1:1,000), and GAPDH (1:000), respectively. After 16 h, the membranes were incubated with goat anti-rabbit (1:1,000, BS13278, Bioworld) or goat anti-mouse (1:1,000, BS13278, Bioworld) secondary antibodies for 2 h at room temperature. The immunoreactive proteins were visualized using a Chemi DocXRS + Imaging System (BioRad). Finally, band intensity was quantified by Image J.

### Statistical Analysis

All data were expressed as mean ± standard error of the mean (SEM). Comparisons of experimental data in different groups were performed using the one-way analysis of variance (ANOVA) followed by Tukey’s test with GraphPad Prism 5 software (GraphPad Software Inc., USA). For all tests, a significant difference was indicated by *p < 0.05, **p < 0.01, and ***p < 0.001.

## Results and Discussion

### Characterization of PEG and SBMA Hydrogels

**FIGURE 1:**
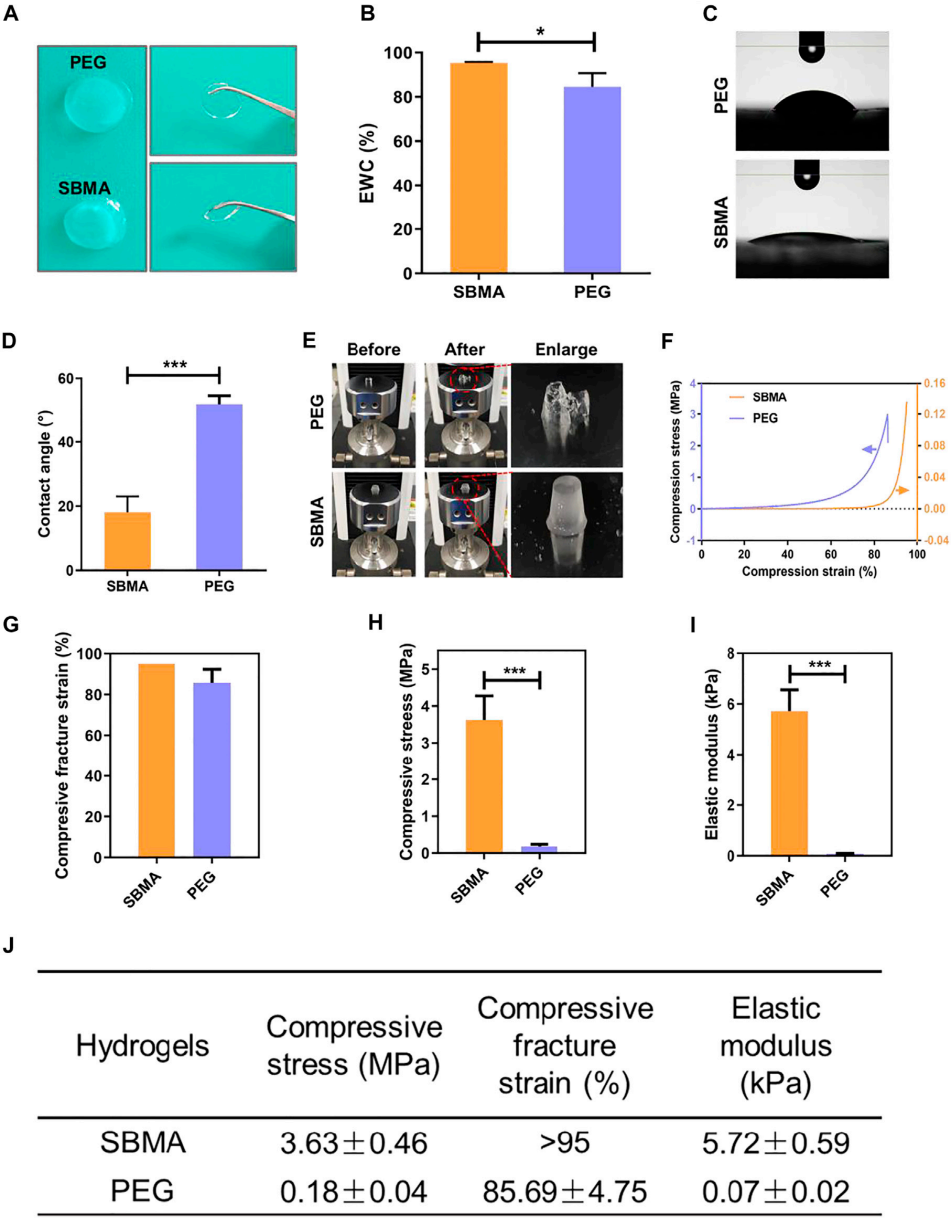
Characterization of PEG and SBMA hydrogels. **(A)** Appearance of PEG and SBMA hydrogels. **(B)** Equilibrium water content (EWC) of PEG and SBMA hydrogels incubated in normal saline. **(C)** Photographs of the contact angle measurements of PEG and SBMA hydrogels. **(D)** Bar diagram of quantified contact angle of PEG and SBMA hydrogels. **(E)** Representative photographs of PEG and SBMA hydrogels before and after loading compression force. **(F)** Compression stress–strain curves of PEG and SBMA hydrogels. **(G)** Bar diagram of the compressive fracture strain. **(H)** Bar diagram of the compressive stress. *n* = 3. **(I)** Bar diagram of the elastic modulus. **(J)** Summary of the mechanical properties of PEG and SBMA hydrogels. Significant difference is indicated as *p < 0.05, ***p < 0.001, and *n* = 3.

The fabrication of PEG and SBMA hydrogels exactly followed our previous reports (Wu et al., 2018; He et al., 2019). It is generally believed that a moist environment is more conducive to wound healing (Korting et al., 2011). Appropriate hydration helps to not only accelerate cell proliferation but also reduce the incidence of infection (Francesko et al., 2018). First, we test the equilibrium water content (EWC) and mechanical property of both PEG and SBMA hydrogels. As shown in Figures 1A,B, both hydrogels showed a smooth and transparent appearance with comparable EWC content. It has been reported that the hydrophilicity of biomaterials has a great implication on cell attachment and growth and is a necessary condition for cell proliferation (Ru et al., 2015; Kurusu and Demarquette, 2019). Therefore, the contact angle of PEG and SBMA hydrogels were further investigated. As shown in Figures 1C,D, the water contact angle of SBMA (18.0 ± 4.1^o^) was significantly lower than that of PEG (51.7 ± 2.2^o^) (***p < 0.001). The lower contact angle of the SBMA hydrogel demonstrated that its hydrophilicity is higher than that of PEG. Next, the mechanical property was further investigated by compression tests of hydrogels, as shown in Figure 1E. After the compression test, the PEG hydrogel was crumbled into fragments, while the SBMA hydrogel retained its initial preload form. The stress–strain curves were further collected in Figure 1F, and the compressive stress, strain, and elastic modules were calculated based on the stress–strain curves (Figures 1G–J). It was observed that the SBMA hydrogel showed a maximum compressive stress of 3.63 ± 0.65 MPa and an elastic modulus of 5.72 ± 0.59 kPa without being broken at strain more than 95%, suggesting the excellent elastic property of the SBMA hydrogel consistent with Figure 1E. By contrast, a fracture occurred in the PEG hydrogel at strain 85.69 ± 6.71% with a maximum compressive stress of 0.18 ± 0.04 MPa and an elastic modulus of 0.07 ± 0.02 kPa, indicating that the PEG hydrogel was very brittle.

### Zwitterionic SBMA Hydrogel Accelerates PU Healing

The PU model of rats established by magnets and the schematic illustration of the model are shown in Supplementary Figure S1. To evaluate the success establishment of the PU model, PU was first compared with the acute wound in wound healing closure on a macroscopic scale, and the pathological changes of PU were observed after H&E staining (Supplementary Figure S2). Different to the acute wound that kept shrinking until it healed completely without the necrotic tissue, PU exhibited a significantly slower wound closure upon wound size and was covered with the necrotic tissue during its regeneration period (14 days, Supplementary Figure S2A). Furthermore, wound healing closure rates at each time of the two groups are summarized and shown in Supplementary Figure S2B. The wound healing rate of PU was lower than that of the acute wound group at each observation time. And on day 14, the closure area of PU (72.5 ± 3.3%) was significantly smaller than that of the acute wound group (98.6 ± 0.2%) that had healed almost completely (***p < 0.001). The results of H&E staining showed that the dermal part of normal skin tissues (Supplementary Figure S2C) was arranged in an orderly manner with complete hair follicles and other skin appendage structures without obvious inflammatory cell infiltration. In contrast, skin tissue structures of PU (the arrows indicate the inflammatory cells) were not integrated with multiple inflammatory cells that appeared in the disordered granulation tissue. All these results indicate that the self-healing ability of PU wound is significantly much slower than that of acute wound.

**FIGURE 2:**
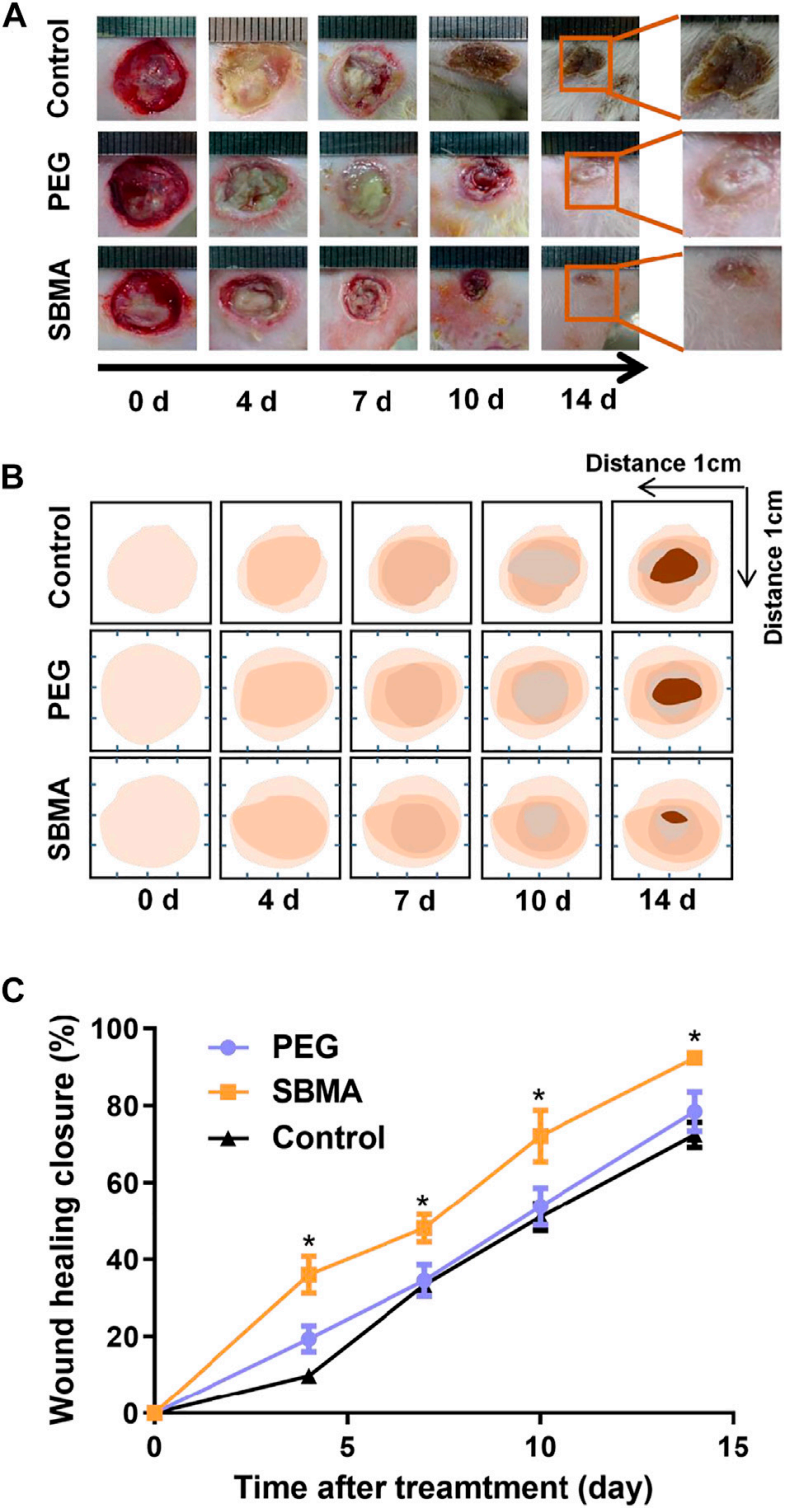
SBMA and PEG hydrogels accelerate healing in pressure ulcers. **(A)** General observation of the wound healing extent on day 0, 4, 7, 10, and 14 after treatments of PEG and SBMA hydrogels. The unit length in the images was 1 mm. **(B)** Schematic diagram that mimicking wound healing process in two groups. **(C)** Statistical summary of the wound healing closure of pressure ulcer in each group. Significant difference is indicated as *p < 0.05, compared to the PEG group, *n* = 3–6.

Zwitterionic SBMA hydrogel has been prove to be effective on wound healing due to its excellent anti-inflammation and angiogenesis performance in our previous work (Wu et al., 2018; Huang et al., 2019). Further *in vivo* experiments were carried out to evaluate the efficacy of SBMA hydrogels on PU healing, in comparison to the PEG hydrogel. In these experiments, SBMA and PEG hydrogels were applied on PU wound to observe the effect of hydrogels on the wound healing process *in vivo*. The wounds without treatment were set as control. The ulcer healing progress was recorded at different times upon hydrogel treatment, during the whole period in Figure 2A, and the schematic diagram of the trace of wound healing closure as shown in Figure 2B. In comparison with the images, the SBMA hydrogel–treated wound recovered relatively faster with a neater appearance than the PEG hydrogel–treated group and the control group. Especially, on the 14th day, a significantly smaller wound site is observed in the SBMA-treated group than in the PEG group with a scab covering the wound (*p < 0.5). Consistent with the macroscopic observation of wound healing in Figures 2A,B, the quantitative closure rate of the SBMA-treated group was always much faster than that of the PEG group and the control group, as shown in Figure 2C. Especially on day 4, the wound recovery rates of the PEG group (18.5 ± 1.9%) were significantly lower than those of the SBMA group (38.3 ± 3.0%) (*p < 0.5). And on day 14, the SBMA-treated group almost healed completely with an average closure rate of 92.3 ± 0.7%. On the contrary, the wounds treated by the PEG hydrogel had quite a similar healing efficacy with that of the control group, indicating the limitation of PEG in promoting PU healing. Taken together, these data verified that the SBMA hydrogel could promote relatively faster PU wound regeneration *in vivo*.

**FIGURE 3:**
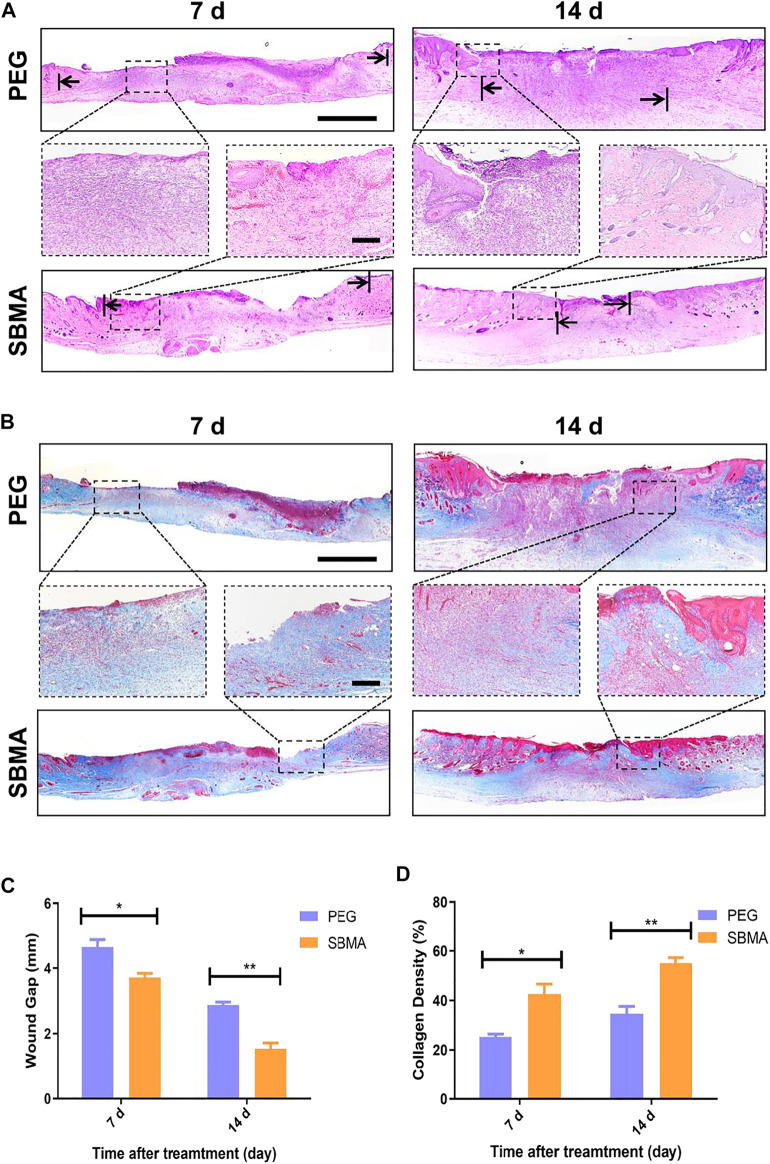
Histological analysis of wound tissue sections on days 7 and 14 post-treatment. **(A)** H&E staining images of skin wounds on days 7 and 14 after the treatments of PEG or SBMA hydrogels. The black arrows indicate the edges of the healing PU wounds. The scale bars are 1 mm **(left)** and 200 μm **(right)**. **(B)** Representative images of Masson’s Trichrome staining of the wounds on day 7 and 14 after different treatments. The scale bars are 1 mm **(left)** and 200 μm (right, magnified images). **(C)** Quantified granulation tissue gap on day 7 and 14. **(D)** Quantified collagen density on day 7 and 14. Significant difference is indicated as *p < 0.05, **p < 0.01, and *n* = 3.

Next, we performed hematoxylin and eosin (H&E) staining and Masson’s Trichrome staining (MTS) to investigate the granulation formation and collagen deposition upon the wound healing process. As shown in Figures 3A, C, on day 7, the PEG group exhibited a large breadth of unhealed wound (the arrows indicate the edges of the healing PU wounds) covered by a scab which was seen in macroscopic photos. By contrast, the wound treated with the SBMA hydrogel (4.6 ± 0.4 mm) had much narrower wound gap than that treated with the PEG group (3.7 ± 0.2 mm) (*p < 0.5). The trend is the same on day 14 (SBMA: 2.9 ± 0.2 mm *versus* PEG: 1.5 ± 0.3 mm). Furthermore, for the SBMA-treated group, thicker and more mature tissue formation with plentiful hair follicles appeared in the wound site (14.9% unhealed) than the PEG hydrogel-treated group with few hair follicles formed upon the wound site (37.3% unhealed). Remodeling of chronic wound contains efficient deposition of ECM component, mainly collagen, the important structural biomolecule. It is proved that slow accumulation of the collagen expression in pressure ulcer wounds was one of the reasons leading to the impaired wound healing process (Jiang et al., 2014). Herein, Masson’s trichrome staining (MTS) was used to detect the collagen deposition in healed wound. Same with H&E results, the SBMA-treated group showed a denser collagen deposition than the PEG group. As shown in Figure 3B, thicker collagen bundles (blue) were found in a more orderly arrangement in wounds treated with the SBMA hydrogel than the wounds treated with the PEG hydrogel. And quantitative data in Figure 3D showed that the wound treated with the SBMA hydrogel possessed more collagen formation than that in the PEG group on both the 7th and 14th days after each treatment. These data again verified that the SBMA hydrogel possessed better capacity in accelerating wound regeneration on a microlevel than the PEG hydrogel.

### Zwitterionic SBMA Hydrogel Promotes Angiogenesis and ECM Reconstruction

As the blood vessels are the key to the wound healing by supporting with necessary oxygen and nutrients, we first investigated the angiogenesis in the PU wound beds after the treatment of PEG and SBMA hydrogel. Specifically, immunohistochemical staining of CD31 and VEGF was performed to evaluate the angiogenesis. CD31 was a marker of vascular endothelial cells, while VEGF was an important growth factor that regulated angiogenesis (Chen Y et al., 2020). The expressions of CD31 and VEGF are highly relevant to the angiogenesis. As shown in Supplementary Figures S3A,B, the number of microvessels (CD31 positive area) in wounds treated by the SBMA hydrogel (8.0 ± 0.7/mm^2^) was higher than that of those in the PEG-treated group (5.0 ± 0.7/mm^2^) (***p < 0.001). In addition, the expression of VEGF in the wounds treated by the SBMA hydrogel (2879.0 ± 244.6/mm^2^) was significantly higher than that of those in the PEG-treated group (1804.0 ± 140.4/mm^2^) (**p < 0.01). To further validate this finding, Western blotting was also applied. Consistent with the result of immunohistochemical staining, the WB result demonstrated that the expression levels of CD31 and VEGF were higher in the SBMA group than those in the PEG group (Supplementary Figures S3C, E). These results indicated that the SBMA hydrogel exhibited better effect toward angiogenesis in PU healing than the PEG hydrogel.

**FIGURE 4:**
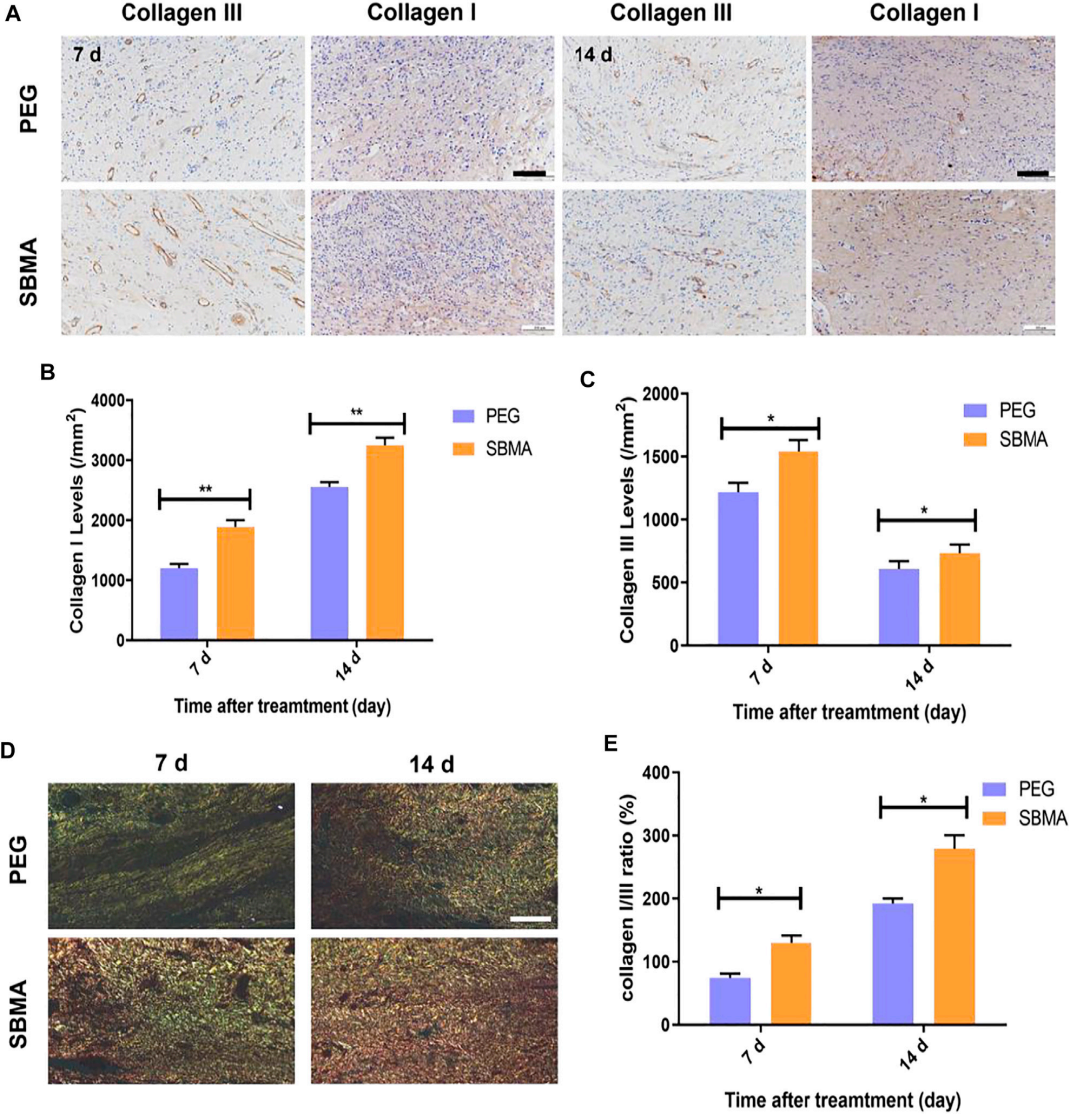
PEG and SBMA hydrogels promote the formation of collagen deposition. **(A)** Immunohistochemical staining results of collagen III (scale bar = 50 µm) and collagen I (scale bar = 100 µm) for wounds treated by PEG or SBMA hydrogels for 7 and 14 days. **(B)** Quantitative analysis of collagen I levels at days 7 and 14 after treating with PEG and SBMA hydrogels. **(C)** Quantitative analysis of collagen III levels at day 7 and 14 after treating with PEG and SBMA hydrogels. **(D)** Representative images of picrosirius red (PSR) staining of collagen fibers at the wound areas on days 7 and day 14 (scale bar = 100 µm). **(E)** Quantitative analysis of collagen I/III ratio at day 7s and 14. Significant difference is indicated as *p < 0.05, **p < 0.01, *n* = 3.

We further investigate the detailed collagen type between the two hydrogel-treated groups. Collagens I and III (Coll I and Coll III) are two main collagens with the highest collagen content in the dermal layer of the skin. As shown in Figure 4A, the expression of Coll I gradually increased during healing for both groups. And more expression of Coll I was observed in wounds treated with SBMA (1953.3 ± 64.9/mm^2^ on day 7 and 3248.0 ± 50.7/mm^2^ on day 14) than in the wounds treated with PEG (1,236.7 ± 56.2/mm^2^ on day 7 and 2555.3 ± 95.6/mm^2^ on day 14) (Figure 4B). Coll I kept increasing during the treatment, while the accumulation of Coll III increased first and then reduced (Figure 4C) by metalloproteinase (Bohn et al., 2016). Quantitatively, Coll III deposition on day 7 (1,218.3 ± 91.8/mm^2^ in the PEG group and 1,543.3 ± 116.2/mm^2^ in the SBMA group) in two groups was markedly higher than that on day 14 (609.3 ± 80.9/mm^2^ in PEG and 733.7 ± 89.9/mm^2^ in SBMA), and more expression of Coll III was detected in the SBMA group than that of the PEG group at both day 7 and day 14. During the wound healing process, Coll III will be slowly degraded by metalloproteinase and the stronger Coll I will gradually be generated instead (Bohn et al., 2016). Therefore, normally Coll I is considered to be mature collagen, while Coll III is more likely to appear in the early stages of wound healing (Cuttle et al., 2005). After picrosirius red staining, the more mature Coll I appeared with red or yellow, while Coll III was green. Figures 4D,E indicated that wound treated with the SBMA hydrogel has a larger Collagen I/III ratio than that treated in the PEG-treated group on both days 7 and 14, suggesting that the SBMA hydrogel triggered quicker maturation of collagen than PEG hydrogel did. These results reveal that in accordance with the wound closure rate, H&E, MTS, and picrosirius red results, SBMA can effectively accelerate the regeneration of PU by not only expressing more collagen deposition on the wound site but also promoting the maturation as well as reconstruction of mature ECM formation.

**FIGURE 5:**
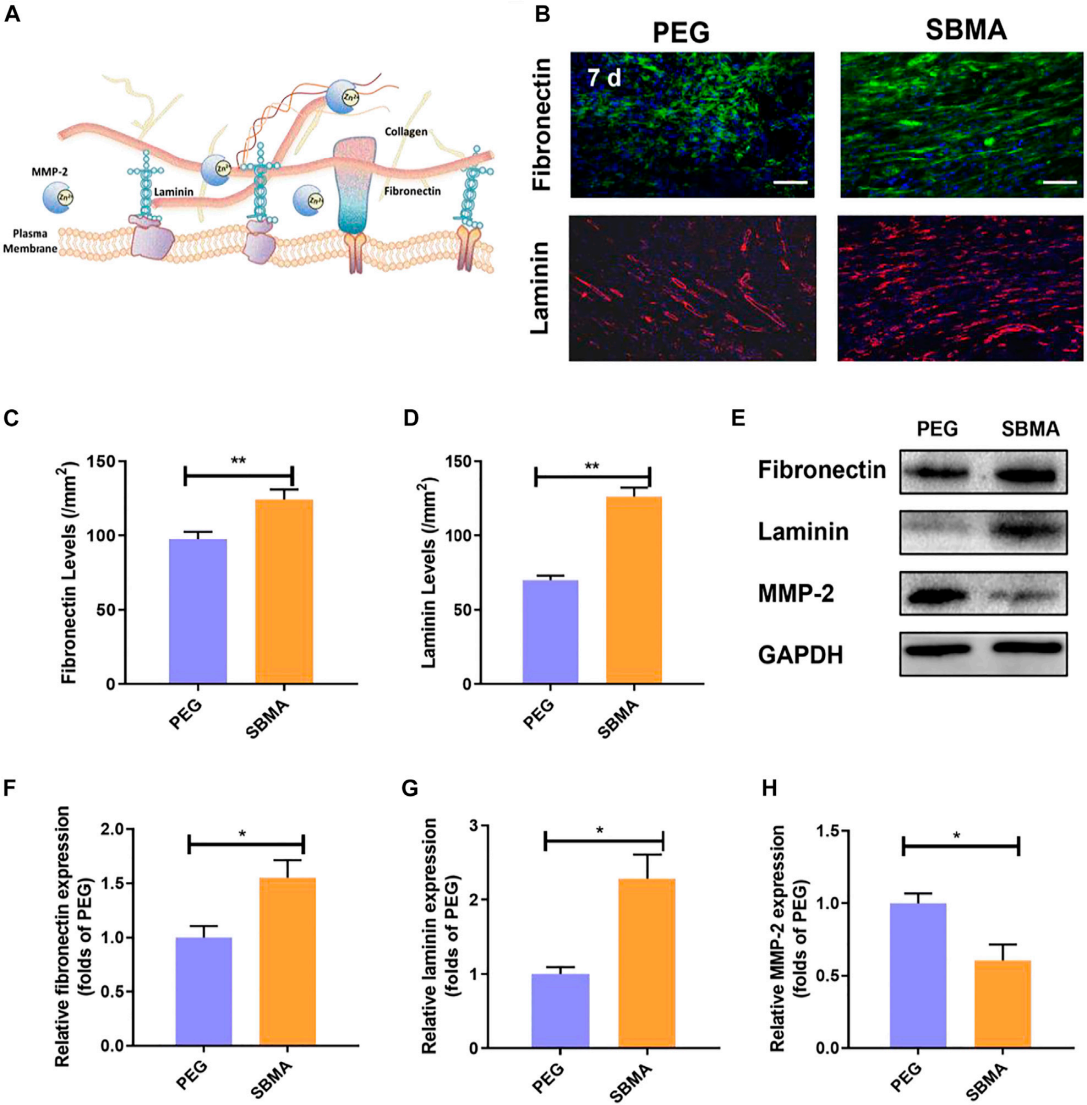
*In vivo* ECM production. **(A)** Scheme of ECM production. **(B)** Immunofluorescent staining of fibronectin (green) and laminin (red) for the two groups on day 7 (scale bar = 50 µm). **(C)** Quantitative fibronectin levels in wounds on day 7 for the two groups. **(D)** Quantitative laminin levels in wounds on day 7 for the two groups. **(E)** Western blotting for fibronectin, and MMP-2 expressions in pressure ulcer of the PEG and SBMA groups. The gels have been run under the same experimental conditions, and cropped blots are used here. **(F–H)** Optical density values of fibronectin, laminin, and MMP-2 were quantified and analyzed each group. Significant difference is indicated as *p < 0.05, **p < 0.01, ***p < 0.001, and *n* = 3.

Furthermore, the deposition of collagen depends strongly on the presence and stability of ECM fibronectin and laminin (Sottile et al., 2007). As fibronectin is present in the early stage of ECM reconstruction, stronger and more stable collagen fibers replace fibronectin during wound remodeling. Fibronectin and laminin, two key ECM-related protein components, are required for epithelial migration and cellular adhesion during the initial hemostasis and granulation tissue formation (Figure 5A) (Wilkinson et al., 2019). As shown in Figure 5B, the treatment of zwitterionic SBMA hydrogel led to increased fibronectin (124.3/mm^2^ vs. 97.6/mm^2^ with **p < 0.01 seen Figure 5C) and laminin deposition (126.3/mm^2^ versus 70.0/mm^2^ with **p < 0.01 seen Figure 5D). To further confirm that zwitterionic SBMA hydrogel can indeed induce more ECM-related protein production, the expression of fibronectin, laminin, and related matrix metalloproteinase MMP-2 proteins was measured using Western blotting in each group. As a family member of zinc-dependent matrix-degrading enzymes, MMP-2 has previously been shown to degrade ECM-related components such as fibronectin and laminin (Jiao et al., 2012). As shown in Figures 5E–H, the expression levels of fibronectin and laminin of the SBMA-treated group significantly increased (*p* < 0.05), whereas the expression level of MMP-2 significantly decreased compared with the PEG group (*p* < 0.05). Wounds from PU have been examined with elevated MMPs, leading to the nonhealing wounds (Yager et al., 1996). Thus, here, based on the observation, we hypothesized that the fibronectin together with laminin upon wound treated by the SBMA hydrogel was upregulated by the inhibition of MMP-2. By reserving MMP-2, SBMA hydrogel would indeed improve the ECM reconstruction upon pressure ulcer through fibronectin augmentation.

### SBMA Hydrogel Activates Autophagy *via* Inhibition of the PI3K/AKT/mTOR Signaling Pathway

**FIGURE 6:**
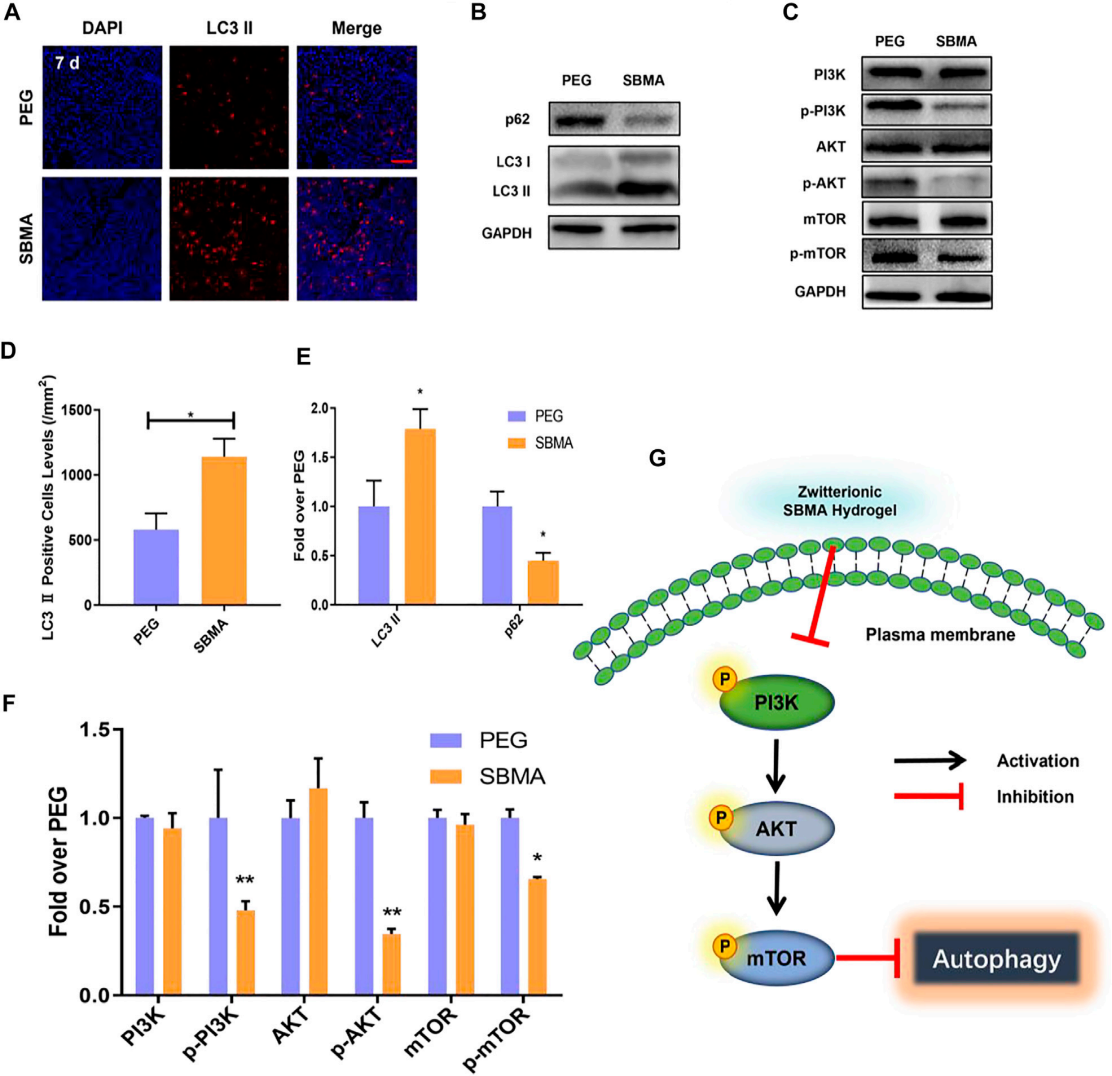
*In vivo* autophagy investigation. **(A)** Immunofluorescent staining of LC3 Ⅱ (red) for the two groups on day 7 (scale bar = 50 µm). **(B–C)** Western blotting for LC3 Ⅱ, P62, PI3K, p- PI3K, AKT, p-AKT, mTOR, and p-mTOR expressions in the pressure ulcer of the PEG and SBMA groups. The gels have been run under the same experimental conditions, and cropped blots are used here. **(D)** Immunohistochemical results with LC3 Ⅱ levels in wounds on day 7 for the two groups. **(E–F)** Optical density values of LC3 Ⅱ, P62, PI3K, p- PI3K, AKT, p-AKT, mTOR, and p-mTOR were quantified and analyzed each group. **(G)** Scheme of zwitterionic SBMA hydrogel treatment inhibited the PI3K/Akt/mTOR signaling pathway and upregulated autophagy. Significant difference is indicated as *p < 0.05, **p < 0.01, ***p < 0.001, and *n* = 3.

Autophagy is one highly conserved process of intracellular degradation of organelles as well as proteins (Li et al., 2015). It is revealed in rat that several autophagy-related proteins have altered during the mechanical compression, which implies the involving of autophagy in pressure ulcer (Teng et al., 2011). However, proper level of autophagy may be beneficial for cell survival by adapting cell toward external compressive stress, while excessive autophagy may lead to cell apoptosis (Scott et al., 2007). Thus, the autophagy may play an important role in cell viability and inflammation in PU beds, which might impact the healing of the wound (King, 2012). As the expression of MMP-2 can be regulated by autophagy, we further investigated the autophagic levels in PU beds by evaluating autophagosomal protein LC3II and autophagic substrate proteipn p62. As shown in Figure 6A, autophagosomes were marked red by LC3II and nuclei were marked blue by DAPI. Immunofluorescence results showed that more autophagosomes were produced in the SBMA-treated group (1,140.33 ± 138.96/mm^2^) than in the PEG group (580.67 ± 124.43/mm^2^) (Figure 6D). Furthermore, the Western blot result of LC3II expression exhibited that the SBMA-treated group was 1.8-fold higher than PEG (Figures 6B,E), which were consistent with immunofluorescence result. Moreover, compared with the PEG group, significantly a lower expression level of autophagic substrate protein p62 was observed in the SBMA group according to the Western blot result with the expression level of 1.00 ± 0.15 for the PEG group while 0.45 ± 0.08 for the SBMA group. Taken together, these observations indicated that the SBMA hydrogel upregulates autophagy in PU.

It is the consensus that PI3K/AKT/mTOR is an important pathway in regulating autophagy (Zhao et al., 2015). As is known, PI3K first activates Akt, which in turn leads to phosphorylation and activation of mTOR (Heras-Sandoval et al., 2014). The mTOR protein is the main factor that regulates the autophagy activity. Its inhibition triggers the dephosphorylation of mTOR and ultimately increases the level of autophagy. To investigate the mechanisms of SBMA-induced autophagy, we performed Western blotting on two groups treated with PEG and SBMA hydrogels. The results from Western blotting showed that the SBMA hydrogel did not alter the expression of the unphosphorylated protein of PI3K, AKT, and mTOR. However, compared with the PEG group, the protein of p-PI3K, p-AKT, and p-mTOR was significantly downregulated after the treatment with the SBMA hydrogel (Figures 6C, F). In conclusion, these data revealed that the mechanism of SBMA hydrogel promoted regeneration of PU is related to the upregulation of autophagy by inhibition of the PI3K/AKT/mTOR signaling pathway as illustrated in Figure 6G.

### SBMA Hydrogel Decreases Inflammation *In Vivo*

**FIGURE 7:**
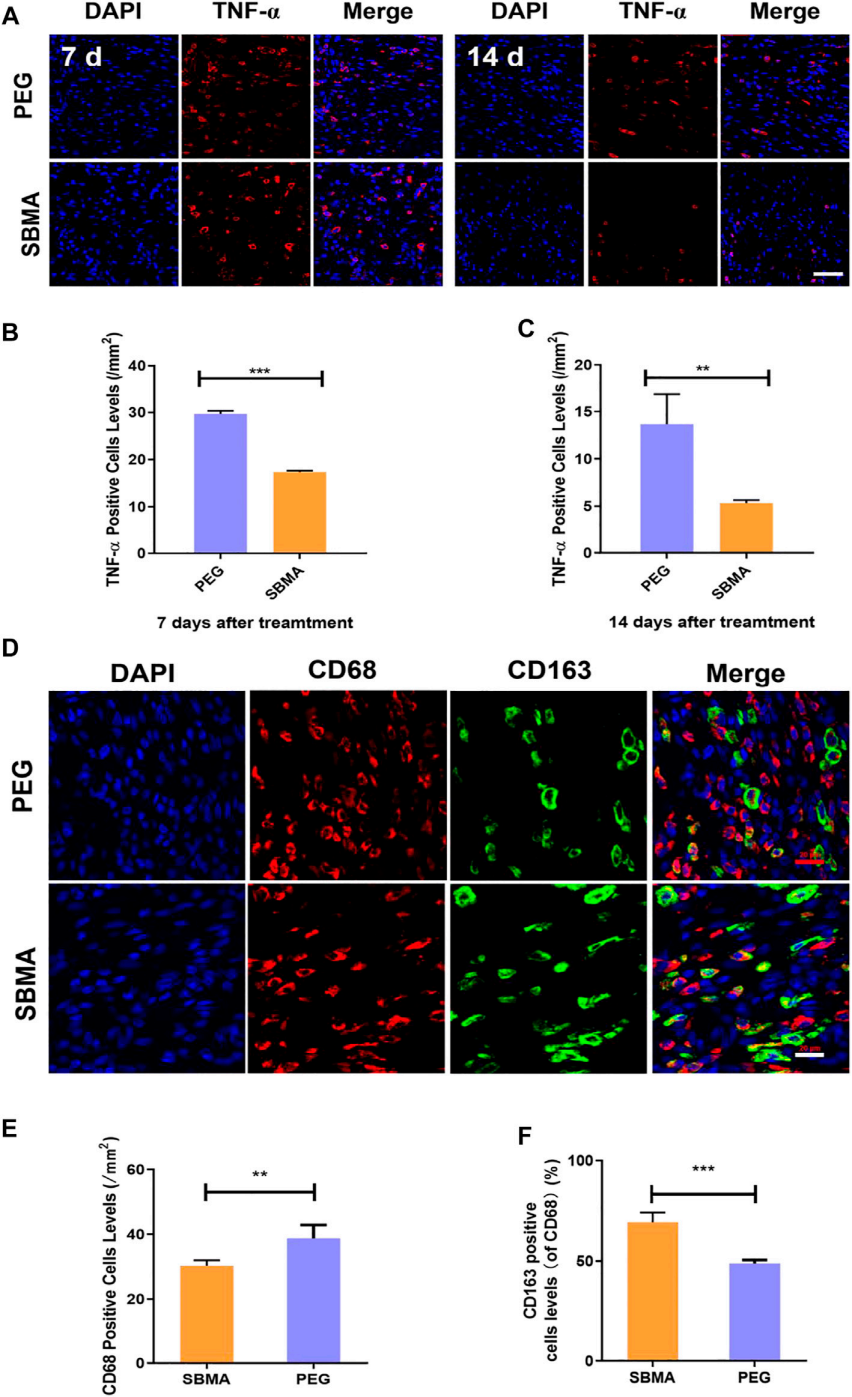
*In vivo* expression of the inflammation factor. **(A)** Immunofluorescent staining of TNF-α for wounds treated by PEG or SBMA hydrogels on day 7 and day 14. Red: TNF-α; blue: DAPI, nuclei. The scale bar was 50 µm. **(B)** Quantified TNF-α–positive cells levels on day 7 at wound sites after the treatments of PEG or SBMA hydrogels. **(C)** Quantified TNF-α–positive cell levels on day 14 at wound sites after the treatments of PEG or SBMA hydrogels. **(D)** Immunofluorescent staining of macrophages. CD68: total macrophage; CD163: M2 phenotype macrophage; DAPI: nuclei (scale bar = 20 μm). **(E)** Bar diagram of the CD68-positive cell level, *n* = 3. **(F)** Bar diagram of the percentage level of CD163-positive cell in comparison to CD68-positive cells. Significant difference is indicated as **p < 0.01, ***p < 0.001, and *n* = 3.

The anti-inflammatory property of SBMA was further assessed due to the non-fouling property of zwitterionic SBMA hydrogel upon PU. Tumor necrosis factor-α (TNF-α) as a pro-inflammatory cytokine is one of the indicators reflecting the inflammatory response (Landén et al., 2016). We investigated the expression of TNF-α upon PU wounds on days 7 and 14 by immunofluorescence staining. As demonstrated in Figures 7A,B, on day 7, more TNF-α–positive cells were observed in the PEG-treated group (29.7 ± 1.0/mm^2^) than the SBMA-treated group (17.3 ± 0.5/mm^2^). On day 14, the number of TNF-α–positive cells in all wounds decreased, and very few were expressed in the SBMA-treated group (5.3 ± 0.6/mm^2^) compared with the PEG-treated group (13.7 ± 5.5/mm^2^) (Figure 7C). This result indicated that zwitterionic SBMA could significantly inhibit inflammation by downregulating the expression of TNF-α due to its excellent nonfouling ability toward the wound site. Therefore, this result further confirmed that zwitterionic SBMA hydrogel played an anti-inflammatory role in promoting PU wound healing by the reducing expression of inflammatory promoter TNF-α.

To further assess the inflammatory level of the pressure ulcers undergoing different treatments, we also investigated the macrophage status in the wound bed. Macrophages can polarize between pro-inflammatory M1 phenotype and anti-inflammatory M2 phenotype, which regulates the inflammation status in the wound area (Ferrante and Leibovich, 2012; Wu et al., 2019). Thus, the macrophage status can reflect the inflammation situation in the wound. Herein, CD68 (a marker for total macrophages) and CD163 (a specific marker for M2 macrophages) were selected for immunofluorescence staining to evaluate the macrophage status in the PU area after the treatment of PEG or SBMA hydrogels. As shown in Figure 7D, CD163-positive cells were less in the PEG group, while CD163-positive cells were more widely distributed in PU treated with the SBMA hydrogel. According to the quantitative results in Figure 7E, the total macrophages in the PEG group (38.80 ± 3.66/mm^2^) were significantly higher than those in the SBMA group (30.25 ± 1.48/mm^2^) (**p < 0.01). Although the number of total macrophages in the SBMA treatment group was less, the ratio of M2-type macrophages to total cells in the SBMA treatment group (69.34 ± 4.34%) was significantly higher than that in the PEG group (48.90 ± 1.52%) (Figure 7F) (***p < 0.001). Therefore, this result further validated that zwitterionic SBMA hydrogel played an anti-inflammatory role in promoting PU wound healing by promoting M2 polarization of macrophages.

## Conclusion

In summary, followed by outstanding healing efficiency of zwitterionic SBMA hydrogel on acute and chronic wounds, this study first comprehensively demonstrated that the SBMA hydrogel could significantly promote wound skin ECM remolding by the upregulation of fibronectin and laminin expression as well as inhibition of MMP-2. Moreover, further study revealed that the inhibition of MMP-2 by the SBMA hydrogel was related to the activation of autophagy. Zwitterionic SBMA hydrogel effectively upregulated autophagy by inhibiting the PI3K/AKT/mTOR signaling pathway. Overall, these findings suggested that the zwitterionic SBMA hydrogel dressing improves PU healing by promoting ECM reconstruction through the downregulation of MMP-2 through the activation of autophagy *via* the PI3K/AKT/mTOR signaling pathway. This work implies that MMP-2 and autophagy may become a future potential target for designing of biomaterials to treat PU.

## Data Availability

The original contributions presented in the study are included in the article/Supplementary Material; further inquiries can be directed to the corresponding authors.
