# Supplementary material for: Zwitterionic Hydrogel Activates Autophagy to Promote Extracellular Matrix Remodeling for Improved Pressure Ulcer Healing
Source: Front Bioeng Biotechnol. 2021 Oct 8;9:740863. doi: 10.3389/fbioe.2021.740863 (PMC8531594; doi:10.3389/fbioe.2021.740863)
Supplement: Supplementary file 1 [file DataSheet1.docx]

Supporting information

**Zwitterionic Hydrogel Activates Autophagy to Promote Extracellular Matrix Remodeling for Improved Pressure Ulcer Healing**

Yuan Li^1,2#^, Shishuang Jiang^2#^, Liwan Song^2^, Zhe Yao^1^, Junwen Zhang^3^, Kangning Wang^2^, Liping Jiang^2^, Huacheng He^3^*, Cai Lin^1^*, Jiang Wu^1,2^*

^1^Department of Burn, The First Affiliated Hospital of Wenzhou Medical University, Wenzhou, Zhejiang, P. R. China, 325035

^2^School of Pharmaceutical Sciences, Key Laboratory of Biotechnology and Pharmaceutical Engineering, Wenzhou Medical University, Wenzhou, Zhejiang, P. R. China, 325035

^3^College of Chemistry and Materials Engineering

Wenzhou University, Wenzhou, Zhejiang, P. R. China, 325035

* Corresponding Authors:

Huacheng He: hehc@wzu.edu.cn

Cai Lin: 13025092850@163.com

Jiang Wu: woody870402@hotmail.com

# These authors contributed equally to this work.

**Figure S1.** (A) Establishment of pressure ulcer (PU) model with magnets. (B) Diagram of PU model establishment. (C) Macroscopic observation on the wound surface of pressure ulcer model after the 1st, 2nd, 3rd and 4th I/R. The unit is 1 mm.

**Figure S2.** Self-healing ability of acute wound (AW) and pressure ulcer (PU) wound. (A) Macroscopic comparison of wound healing between AW and PU. The unit length is 1 mm. (B) Wound healing closure of AW and PU over the course of healing process, *** *P* < 0.001. (C) Hematoxylin and eosin staining (H&E) images of skin and muscle from normal and pressure ulcer tissues. The red arrows indicate the inflammatory cells. Scale bar = 200 µm.


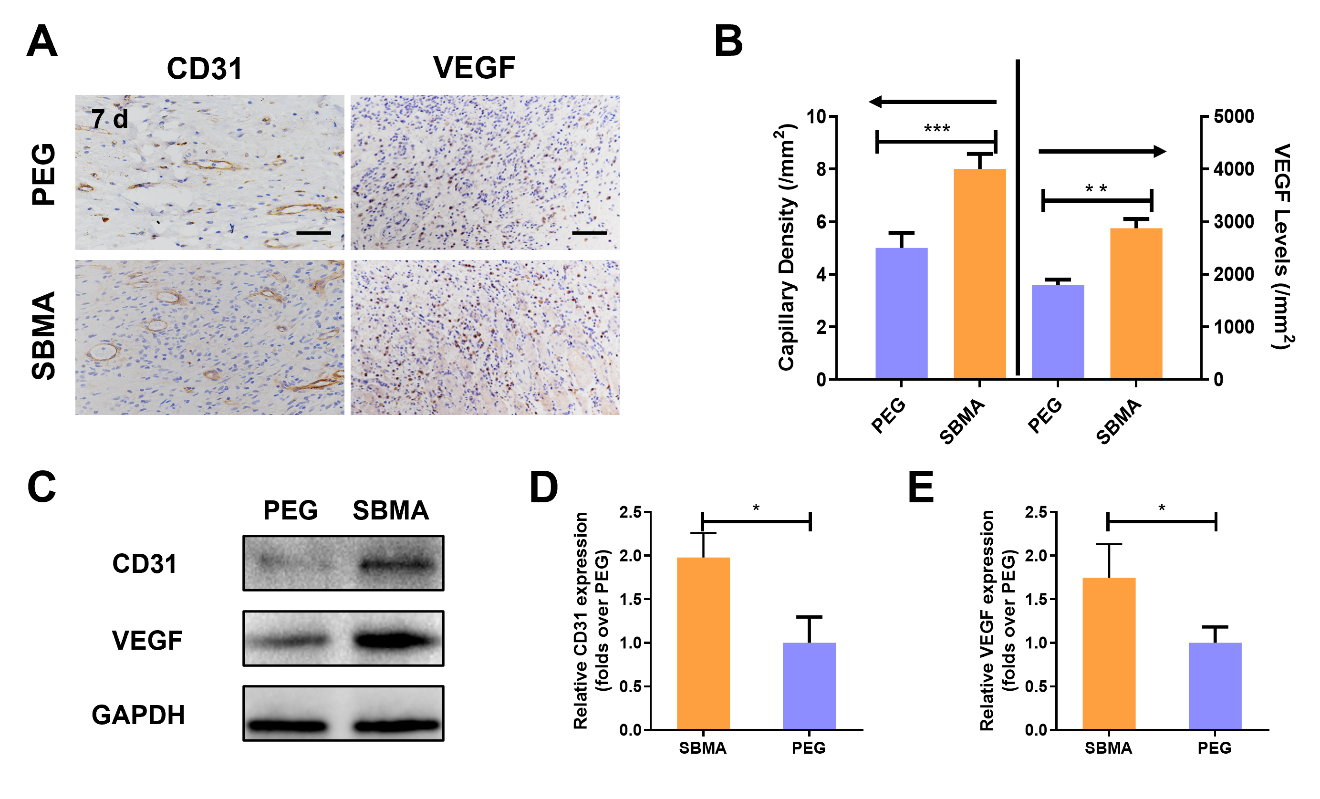


**Figure S3**. PEG and SBMA hydrogels promote angiogenesis. (A) Immunohistochemical results with CD31 (scale bar = 25 µm) and VEGF (scale bar = 50 µm) in PU wound beds on day 7 after the treatments of PEG and SBMA hydrogels. (B) Quantified capillary density and VEGF positive cells levels on day 7 after different treatments. (C) Western blotting for CD31 and VEGF expressions in the PU beds after the treatments of the PEG and SBMA hydrogels. (D-E) Bar diagram of the expressions of CD31 and VEGF (folds to PEG level) in the PU wound beds after different treatments. * *P* < 0.05, ** *P* < 0.01, ****P* < 0.001, n = 3 per group.
